# Diverse Cone-Snail Species Harbor Closely Related *Streptomyces* Species with Conserved Chemical and Genetic Profiles, Including Polycyclic Tetramic Acid Macrolactams

**DOI:** 10.3389/fmicb.2017.02305

**Published:** 2017-11-24

**Authors:** Michelle Quezada, Cuauhtemoc Licona-Cassani, Pablo Cruz-Morales, Angela A. Salim, Esteban Marcellin, Robert J. Capon, Francisco Barona-Gómez

**Affiliations:** ^1^Institute for Molecular Bioscience, The University of Queensland, Brisbane, QLD, Australia; ^2^Evolution of Metabolic Diversity Laboratory, Unidad de Genómica Avanzada (Langebio), Cinvestav-IPN, Irapuato, Mexico; ^3^Australian Institute for Bioengineering and Nanotechnology, The University of Queensland, Brisbane, QLD, Australia

**Keywords:** *Streptomyces*, cone snails, natural product, phylogenomics, polycyclic tetramic acid macrolactams (PTMs)

## Abstract

*Streptomyces* are Gram-positive bacteria that occupy diverse ecological niches including host-associations with animals and plants. Members of this genus are known for their overwhelming repertoire of natural products, which has been exploited for almost a century as a source of medicines and agrochemicals. Notwithstanding intense scientific and commercial interest in *Streptomyces* natural products, surprisingly little is known of the intra- and/or inter-species ecological roles played by these metabolites. In this report we describe the chemical structures, biological properties, and biosynthetic relationships between natural products produced by *Streptomyces* isolated from internal tissues of predatory *Conus* snails, collected from the Great Barrier Reef, Australia. Using chromatographic, spectroscopic and bioassays methodology, we demonstrate that *Streptomyces* isolated from five different *Conus* species produce identical chemical and antifungal profiles – comprising a suite of polycyclic tetramic acid macrolactams (PTMs). To investigate possible ecological (and evolutionary) relationships we used genome analyses to reveal a close taxonomic relationship with other sponge-derived and free-living PTM producing *Streptomyces* (i.e., *Streptomyces albus*). In-depth phylogenomic analysis of PTM biosynthetic gene clusters indicated PTM structure diversity was governed by a small repertoire of genetic elements, including discrete gene acquisition events involving dehydrogenases. Overall, our study shows a *Streptomyces*-*Conus* ecological relationship that is concomitant with specific PTM chemical profiles. We provide an evolutionary framework to explain this relationship, driven by anti-fungal properties that protect *Conus* snails from fungal pathogens.

## Introduction

Polycyclic tetramic acid macrolactams (PTMs) are widespread natural products produced by members of the phyla Actinobacteria and Proteobacteria (class gammaproteobacteria). PTM-producing bacteria have been isolated from complex biological systems, such as sponges, plants and insects (Shigemori et al., 1992; Jakobi and Winkelmann, 1996; Nakayama et al., 1999; Yu et al., 2007; Blodgett et al., 2010). Examples of PTMs produced by symbiotic bacteria include alteramide A produced by *Alteromonas* sp., isolated from the marine sponge *Halichondria okadai* (Shigemori et al., 1992), maltophilin produced by *Stenotrophomonas maltophilia*, isolated from the rhizosphere of rape plants (Jakobi and Winkelmann, 1996), xanthobaccins produced by *Stenotrophomonas* sp. strain SB-K88, isolated from the root of sugar beet (Nakayama et al., 1999), and frontalamides produced by *Streptomyces* sp. SPB78, isolated from the southern pine beetle (Blodgett et al., 2010). These observations suggest that PTMs play important biological and ecological roles yet-to-be described.

Structurally, PTMs are composed of a polycyclic carbocycle (*5-5, 5-5-6 or 5-6-5 ring system*), a macrolactam core, and a tetramic acid moiety (Zhang et al., 2016). Functional analysis using the host *Escherichia coli* for heterologous expression, and *in vitro* reconstitution of individual functional polyketide (PKS) and non-ribosomal synthetase (NRPS) enzymes, have determined that the tetramate polyene precursor is produced via a hybrid PKS/NRPS, whereas the PKS domain is responsible for the PTM backbone assembly (Li et al., 2014) and the NRPS module incorporates L-ornithine (Blodgett et al., 2010). In addition, the biosynthetic pathways of several PTMs (i.e., frontalamides, ikarugamycin, and dihyrodromaltophilin – also known as heat-stable antifungal factor HSAF) have been elucidated using different *in vivo* and *in vitro* approaches (Yu et al., 2007; Halo et al., 2008; Blodgett et al., 2010; Lou et al., 2011; Antosch et al., 2014; Li et al., 2014; Zhang et al., 2014; Greunke et al., 2017). For instance, the six-gene (*ftd*A-F) frontalamide biosynthetic gene cluster (BGC), identified by gene knock out of the *ftd* biosynthetic cluster in *Streptomyces* sp. SPB78, incorporates a sterol desaturase-like enzyme, iterative hybrid PKS/NRPS, two desaturase-like enzymes, a zinc dependent alcohol desaturase and a cytochrome P450 enzyme (Blodgett et al., 2010). The biosynthesis of ikarugamycin from *Streptomyces* sp. was elaborated through heterologous expression of three main biosynthetic components, the iterative hybrid PKS/NRPS enzyme, phytoene desaturase-like enzyme and alcohol dehydrogenase encoded by *ikaA*, *ikaB*, and *ikaC* genes, respectively (Antosch et al., 2014; Zhang et al., 2014; Greunke et al., 2017). Finally, dihydromaltophilin biosynthesis was described in *Lysobacter enzymogenes* strains a decade ago through gene disruption, *in vitro* biochemical assays and heterologous expression experiments (Yu et al., 2007; Lou et al., 2011; Xie et al., 2012). The dihydromaltophilin gene cluster is formed by an iterative hybrid PKS/NRPS, sterol desaturase-like enzyme, ferredoxin reductase, alcohol dehydrogenase and three FAD-dependent oxidoreductase like enzymes.

Cone snails are marine molluscs that comprise the large genus *Conus*, with currently over 800 species identified (Costello et al., 2013). Queensland, Australia, represents a biodiversity hotspot for these molluscs with 133 of 166 Australian species. Cone snails prey on fish, worms or other snails using venoms composed mainly of small peptides (<5 kDa) known as conotoxins or conopeptides. Conotoxins are chemically stable ribosomally synthesized and post-translationally modified peptides that possess high specificity against diverse neuronal targets, many of which are implicated in human diseases (Cruz et al., 1985; Vetter and Lewis, 2012; Inserra et al., 2013). Given the wide range of potential applications of conotoxins as pharmaceutics, cone snails have proved to be an excellent source of molecules for drug discovery and development (Adams et al., 1999; Halai and Craik, 2009). An example of this is the conopeptide marketed under the name Prialt, used clinically as a pain therapeutic (Miljanich, 2004). Previous studies on cone snail-associated bacteria as producers of bioactive metabolites reported neuroactive metabolites (Lin et al., 2011, 2013).

In the current study, we explored the biosynthetic repertoire of five cone snail-derived *Streptomyces* isolated from cone snail specimens collected from the Lady Musgrave Island, Great Barrier Reef, Australia. The observation that several *Streptomyces* isolates displayed common phenotypic colony morphology when grown under specific culture conditions, suggested a taxonomic bias, and perhaps a common (ecological-symbiotic) relationship. This prompted us to conduct a chemical and phylogenomic investigation, in which we confirmed that all isolates were taxonomically related, and shared similar chemical and biological (i.e., antifungal) profiles. A phylogenomic approach was used to establish direct correlations between sequence phylogeny, the presence/absence of enzymes within the PTM BGCs, and the associated natural products. Overall, this report demonstrates a specific association between a discrete lineage of *Streptomyces* and cone snails, and provides an evolutionary framework for further investigations into the biosynthesis of PTMs.

## Materials and Methods

### Cone Snail Collection, Dissection and Microbial Isolation

Cone snails and microorganisms were manipulated under sterile conditions provided by a LabTech class II biological safety cabinet and incubated in either a MMM Friocell incubators (Lomb Scientific, NSW, Australia) or an Innova 42R incubator shaker (John Morris, NSW, Australia) with temperature set at 26.5°C. Cone snail-derived *Streptomyces* were isolated from the stomach and hepatopancreas of five *Conus* species collected in 2011 from the Lady Musgrave Island at the Great Barrier Reef. Freshly collected cone snail specimens were transported in local seawater to the laboratory. The cone snail taxonomy was determined based on an informed observation of the physical characteristics including color of the body, cyphon and outer shell (Röckel et al., 1995) and according to the taxonomic keys available at the *Conus* biodiversity website^1^. Specimens were dissected and tissue was homogenized using a mortar and pestle, homogenate was serial diluted and applied to ISP-4 agar plates (Bacto DIFCO, Cat No. 277210), sealed with parafilm and incubated for 3–4 weeks.

### Taxonomy of *Streptomyces* Isolates

A pure culture of *Streptomyces* isolates obtained by single colony serial transfer on agar plates was cryopreserved at -80°C in 20% aqueous glycerol. Five *Streptomyces* isolates: CMB-CS038, CMB-CS145, CMB-CS143, CMB-CS132, CMB-CS138 were obtained from five distinct species of cone snails, namely *Conus miles, C. ebraeus, C. flavidus, C. coronatus*, and *C. emaciatus*, respectively. The isolates initially formed a white colony, with gray spores after 10 days of incubation at 26.5°C. Genomic DNA from all the isolates was extracted from liquid cultures using the DNAeasy Plant Mini Kit (QIAGEN) as per the manufacturer’s protocol. Bacterial taxonomic identification was performed by rRNA amplification. The rRNA genes were amplified by PCR using the universal primers 27F (5′- AGAGTTTGATCMTGGCTCAG-3′) and 1498R (5′-TACGGYTACCTTGTTACGACTT-3′) purchased from Sigma–Aldrich. The PCR mixture (50 μL) contained genomic DNA (1 μL, 20–40 ng), four deoxynucleoside triphosphates (dNTP, 200 μM each), MgCl_2_ (1.5 mM), primer (0.3 μM each), 1 U of *Taq* DNA polymerase (Fisher Biotec) and PCR buffer (5 μL, 10×). PCR was performed as follows: initial denaturation at 95°C for 3 min, 30 cycles in series of 94°C for 30 s (denaturation), 55°C for 60 s (annealing) and 72°C for 60 s (extension), followed by one cycle at 72°C for 6 min. The PCR products were purified with PCR purification kit (QIAGEN) and sequenced. 16S DNA sequence (∼1200 bp) was used as a query to BLAST against NCBI GenBank available database.

### General Experimental Procedures

Analytical grade solvents were used for both liquid and solid phase extractions (SPE). Spectrophotometric-grade solvents were used for UV and chiroptical measurements. Deuterated solvents were purchased from Cambridge Isotopes (Andover, MA, United States). Specific optical rotations ([α]_D_) were measured on a JASCO P-1010 polarimeter in a 100 mm × 2 mm cell at room temperature. CD spectra were recorded at 22°C on a JASCO J-810 spectropolarimeter. Liquid chromatography-diode array-mass spectrometry (HPLC-DAD-MS) data were acquired on an Agilent 1100 series separation module equipped with an Agilent 1100 series HPLC/MSD mass detector and diode array multiple wavelength detector. Semi-preparative and preparative HPLCs were performed using Agilent 1100 series HPLC instruments with corresponding detectors, fraction collectors and software inclusively. Pure compounds or fractions eluting from semi-preparative and preparative HPLCs were dried on a Christ freeze dryer. Nuclear magnetic resonance (NMR) spectra were acquired on a Bruker Avance 600 MHz spectrometer with either a 5 mm PASEL 1H/D-13C Z-Gradient probe or 5 mm CPTCI 1H/19F-13C/15N/DZ-Gradient cryoprobe, controlled by TopSpin 2.1 software. In all cases spectra were acquired at 25°C (unless otherwise specified) in solvents as specified above, with reference to residual ^1^H or ^13^C signals in the deuterated solvents. Electrospray ionization mass spectrometry (ESIMS) experiments were carried out on an Agilent 1100 series LC/MSD (quadrupole) instrument in both positive and negative modes. High-resolution ESIMS spectra were obtained on a Bruker micrOTOF mass spectrometer either by direct injection in MeCN at 3 μL/min using sodium formate clusters as an internal calibrant.

### Cultivation of Microbes and Chemical Analysis

*Streptomyces* sp. CMB-CS038, CMB-CS145, CMB-CS143, CMB-CS132, CMB-CS138 were cultivated for approximately 15 days in a petri dish (10 cm) containing ISP-4 agar (Burlington, NC, United States). The ISP-4 agar was extracted with 3:1 EtOAc:MeOH (30 mL) and the organic phase concentrated *in vacuo* to yield approximately 10 mg of crude extract. A solution of crude extract prepared in MeOH (1 mg/mL) was subjected to HPLC-DAD-ESI(±)MS analysis (Zorbax SB-C_8_ column, 150 mm × 4.6 mm column, 5 μm, 1 mL/min gradient elution from 90% H_2_O/MeCN to 100% MeCN over 15 min, with constant 0.05% formic acid modifier). *Streptomyces* sp. CMB-CS038 was also cultivated in other media: Marine agar (Becton Dickinson, Franklin Lakes, NJ, United States), Nutrient Agar (Becton Dickinson, Franklin Lakes, NJ, United States) and R2A agar (Becton Dickinson, Franklin Lakes, NJ, United States) to explore the secondary metabolite production capability of this strain.

### Purification and Identification of PTMs from CMB-CS038

A seed culture of *Streptomyces* sp. CMB-CS038 was used to inoculate ISP-4 agar (400 plates), which were incubated at 26.5°C for 10 days, after which the agar was diced and extracted EtOH:MeOH 3:1 (2 L × 1.5 L). The solvent was concentrated *in vacuo* at 40°C and dried under N_2_ at 40°C to yield a crude extract (820 mg). The crude extract was triturated into hexane (150 mg) and MeOH (650 mg) solubles, with the latter subjected to sequential fractionations by C_8_ SPE (30% H_2_O/MeCN to 100% MeCN) and semi-preparative HPLC purification (Zorbax-SB C_18_ column 250 μm × 9.4, 5 μm, 3 mL/min, gradient elution from 10% H_2_O/MeCN to 100% MeCN, with constant 0.01% TFA modifier, over 40 min) to afford dihydromaltophilin.

### Antibiotic Assays

Activities were measured against Gram-positive bacterium *Staphylococcus aureus* ATCC 25923, Gram-negative bacteria *E. coli* ATCC 25922 and *Pseudomonas aeruginosa* ATCC 27853 and a fungus *Candida albicans* ATCC 90028 by the broth micro-dilution method. The test was performed (in triplicate) in 96-well microtiter plates by serial dilution in tryptic soy broth for bacteria and Sabouraud broth for fungi, respectively. Test compounds were prepared and serially (10-fold) diluted in 10% DMSO. An aliquot (20 μL) of each dilution was transferred to a 96-well microtiter plate, followed by freshly prepared microbial broth (180 μL, 10^4^–10^5^ cfu/mL cell density) to give a final test compound concentration ranging from 32 to 0.125 μg/mL. For crude extracts, an aliquot was prepared from dried extract at a concentration of 1 mg/mL (2020 μL). Assay plates were incubated at 37°C for 24 h for bacteria and at 26.5°C for 48 h for yeast. The optical density of each well was measured at 600 nm using a microtitre plate spectrophotometer (POLARstar Omega plate, BMG LABTECH, Offenburg, Germany). The minimum inhibitory concentration (MIC_50_) was determined as the lowest concentration of a test compound that inhibits 50% of microorganism growth. Broth medium with and without microbial inoculation were used as negative controls. The MIC_50_ (μM) for positive control tetracycline against *Staphylococcus aureus* ATCC 25923 was 0.26 μM and for Gram-negative bacteria *E. coli* ATCC 25922 was 0.12 μM and *Pseudomonas aeruginosa* ATCC 27853 was 0.26 μM. The MIC_50_ for positive control ketoconazole against *Candida albicans* ATCC 90028 was 0.22 μM.

### Cytotoxicity Assays

Activity was measured using a MTT [3-(4,5-dimethylthiazol-2-yl)-2,5-diphenyltetrazolium bromide] assay modified from that previously described (Carmichael et al., 1987) using adherent NCI-H460 (human lung carcinoma), SW-620 (human colorectal adenocarcinoma) cells. Briefly, cells were harvested with trypsin and dispensed into 96-well microtitre assay plates at 2,000 cells/well and incubated for 18 h at 37°C with 5% CO_2_ (to allow cells to attach). Test compounds were dissolved in 5% DMSO in PBS (*v*/*v*) and aliquots (20 μL) were tested over a series of final concentrations ranging from 10 nM to 30 μM. Control wells were treated with 5% aqueous DMSO. After 68 h incubation at 37°C with 5% CO_2_, an aliquot (20 μL) of MTT in PBS (4 mg/mL) was added to each well (final concentration of 0.4 mg/mL), and the microtitre plates incubated for a further 4 h at 37°C with 5% CO_2_. After this final incubation the medium was aspirated and precipitated formazan crystals dissolved in DMSO (100 μL/well). The absorbance of each well was measured at OD_580__nm_ at r.t. on a POLARstar Omega microtitre plate reader. IC_50_ values were calculated using Prism 5.0 (GraphPad Software Inc., La Jolla, CA, United States), as the concentration of analyte required for 50% inhibition of cancer cell growth (compared to negative controls). All experiments were performed in duplicate. Vinblastin was used as a positive control for the MTT assay showing an IC50 value of 0.05 μM for NCI-H460 lung cancer cell line and 0.01 μM for SW-620 colon cancer cells.

### Genome Sequencing, Annotation and Mining

The genomic DNA of the isolated strains was sequenced using the MiSeq Illumina platform in the 250 bases paired-end format at the Ramaciotti Centre (Sydney, NSW, Australia). The reads obtained were trimmed using Trimmomatic v0.32 (Bolger et al., 2014) and assembled using Velvet v1.2.10 (Zerbino and Birney, 2008), *K*-mers ranging from 31 to 171 (increasing 10 units per iteration) were tested and the largest assemblies in the lowest number of contigs were selected. RAST (Aziz et al., 2008) and antiSMASH version 3 (Weber et al., 2015) were used for genome annotation.

### Phylogenomic Analysis of PTMs Biosynthesis

For the phylogenomic analysis of PTMs, their BGCs were mined from a genomes database including the genomes of our isolates, actinobacterial genomes selected for their taxonomic distribution and genomes from non-actinobacteria species reported to produce PTMs (Zhang et al., 2016). The sequences were retrieved from GenBank. PTM BGCs were analyzed using the CORASON-BGC pipeline (CORe Analysis of Synthenic Orthologs of Natural products BGCs^2^) and the ikarugamycin BGC as deposited in MIBiG database (accession number: BGC0001435) (Medema et al., 2015) as query. First, we searched for IkaB homologs using BlastP (*e*-value 0.000001, bitscore 1000). Second, neighborhoods of 15 genes upstream and downstream *ikaB* were obtained from the genomes previously annotated using RAST (Aziz et al., 2008). Finally, the selected gene neighborhoods were analyzed using bidirectional best-hits against the ikarugamycin BGC to identify further syntenic homologs. The gene neighborhoods sharing at least three syntenic homologs with *E*-values of less than 1E-6 were considered orthologous PTM BGCs. The amino acid sequences of the homologs of the NRPS-PKS (IkaA) and the beta subunit of the RNA polymerase (rpoB) from selected genomes were identified with BlastP (*e*-value 0.00000001 for both, bitscore 4000 for *ikaA* and 200 for rpoB) extracted, aligned, trimmed and the resulting matrix was used for phylogenetic reconstruction with MrBayes v.3.2.6 (Huelsenbeck et al., 2001) using a mixed substitution model for a million generations. This phylogenetic tree was used to order the representation of the BGCs analyzed and obtained with CORASON-BGC.

## Results

### Isolation of *Streptomyces* spp. from Marine Cone Snails

**FIGURE 1:**
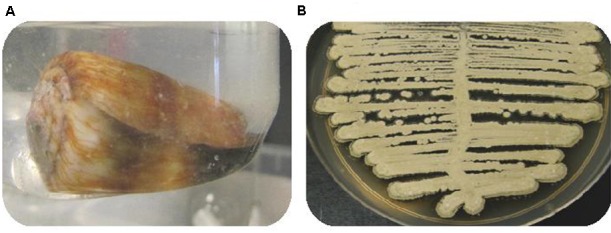
**(A)** Image of cone snail *C. miles* **(B)** ISP-4 agar plate cultivation displaying the common phenotype of *Streptomyces* sp. after 10 days incubation at 26.5°C.

Five venomous cone snail specimens were collected and transported from the Great Barrier Reef to the microbiology laboratory, in local ocean water at room temperature (∼26°C). Detailed morphological examinations indicated that the collected cone snail specimens belonged to three different taxonomic groups, each one belonging to a different species, namely, *C. emaciatus* and *C. flavidus* (*Virgiconus* group), *C. miles* (*Rhizoconus* group) and *C. coronatus* and *C. ebraeus* (*Virroconus* group). A total of 150 microbial strains were isolated from dissected cone snail tissues (venom duct, stomach foot and hepatopancreas) using ISP-4 solid media. Environmental sampling (rock surface areas and organisms found near the cone snails such as sea stars and sea gherkins) during cone snail collection ruled out a plausible environmental contamination given the lack of growth of *Streptomyces-*like organisms. We also found that isolation of PTM producing strains was tissue independent, as all strains were obtained from different organs of the snail (Supplementary Table S1). Most of the microbial isolates (70%) were non-sporulating unicellular bacteria while the rest were spore-forming bacteria, and only one fungus. Based on microscopic and macroscopic phenotypes (e.g., hyphae forming, pigmentation, formation of aerial mycelium and sporulation) (**Figure 1**), we selected a total of five *Streptomyces* isolates to explore their metabolic potential, taking into consideration that all the selected strains were derived from different cone snails. For simplicity, we assigned the following isolation codes: CMB-CS038 (*C. miles*), CMB-CS145 (*C. flavidus*), CMB-CS132 (*C. emaciatus*), CMB-CS143 (*C. coronatus*), and CMB-CS138 (*C. ebraeus*).

### Initial Metabolic Profiling Shows That All Cone Snail-Associated *Streptomyces* spp. Produce a Similar Subset of PTMs

**FIGURE 2:**
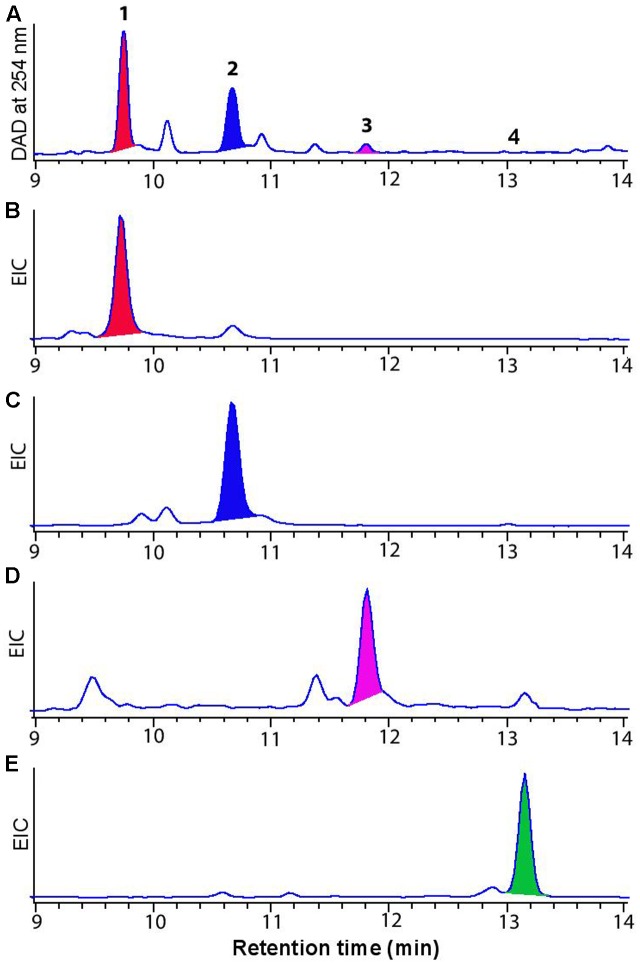
**(A)** HPLC-DAD-MS (254 nm) chromatogram of cone snail-associated *Streptomyces* sp. extracts cultivated on ISP-4 agar **(B)** Single ion extraction (SIE) at m/z 511 [M-H]- corresponding to dehydromaltophilin **(1)** **(C)** at m/z 509 [M-H]- corresponding to **2**, **(D)** at m/z 493 [M-H]- corresponding to xanthobaccin **(3)**, and **(E)** at m/z 491 [M-H]- corresponding to FI-3 **(4)**.

*Streptomyces* strains were first cultured on ISP-4 solid media. After 240 h, extracellular metabolites were extracted using 3:1 MeOH:EtOAc, then dried *in vacuo*, resuspended in MeOH, and analyzed by HPLC-DAD-MS. Initial HPLC-DAD-MS chromatograms showed identical chemical profiles for all five different strains over retention time (*t*_R_) 8.0 to 13.0 min, inclusive of common UV-vis chromophores (λ_max_ 220 and 323) and ESI-MS *m/z* ions (*m/z* 511 [M-H]^-^, *m/z* 509 [M-H]^-^, *m/z* 493[M-H]^-^ and *m/z* 491 [M-H]^-^) (**Figure 2**). Additionally, the same solvent extracts also exhibited antifungal and cytotoxic properties when tested against *Candida albicans* and two human cancer cell lines (SW-620 and NCI-H460) (data not shown).

### Identification and Biological Activity of PTMs Produced by Cone Snail-Associated *Streptomyces* Isolates

**FIGURE 3:**
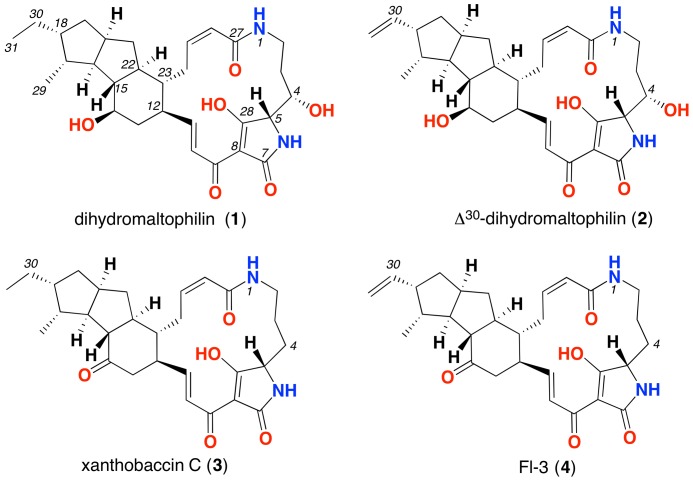
Structure of PTMs produced by cone snail-associated *Streptomyces* spp.

We evaluated *Streptomyces* CMB-CS038 on four different media (ISP-4 agar, marine agar, nutrient agar and R2A agar) and detected significant production of PTMs only on ISP-4 agar. In order to elucidate the chemical structures of the target PTMs, we scaled up the cultivation (400 × ISP-4 petri plates). The resulting extract was fractionated by semi-preparative HPLC to afford PTM 1. Comparison of the NMR (Supplementary Table S2 and Figures S2–S4) and ECD data (Supplementary Figure S5) for **1** with the literature data confirm it to be identical to dihydromaltophilin (Graupner et al., 1997), the absolute configuration of which was solved in 2015 (Xu et al., 2015). The yield of three minor PTMs **2**–**4** co-metabolites (**Figure 2**) was not sufficient for NMR spectroscopic analysis, however, HPLC-DAD-MS analysis using single ion extraction (SIE) and HPLC co-injection with authentic standards confirmed the presence of xanthobaccin C (**3**) (Hashidoko et al., 2000) and the frontalamide precursor FI-3 (**4**) (Blodgett et al., 2010) (**Figure 3**). We sourced authentic standards xanthobaccin C and frontalamide intermediate FI-3 and confirmed their structures by *de novo* spectroscopic analysis, and comparison with literature data (Blodgett et al., 2010). We determined that **2** is new to the literature, and based on the molecular formula and biosynthetic grounds, we tentatively assign the structure as Δ^30^-dihydromaltophilin. We attribute common absolute configurations to co-metabolites (**1**–**4**). Due to limited material, biological assays were only performed for dihydromaltophilin (**1**), which showed antifungal activity against *Candida albicans* (IC_50_ 3 μM) and cytotoxicity against human colon (SW-620, IC_50_ 3.0 μM) and lung (NCI-H460, IC_50_ 5 μM) carcinoma cells (Supplementary Figures S7, S8). See the section “Discussion” (below) for commentary on ambiguities, redundancies and limitations in PTM trivial nomenclature and structure assignments.

### Cone Snail-Associated *Streptomyces* Are Taxonomically Related, Have Reduced Genomes and Conserved PTM BGCs

The observation that different cone snail-associated *Streptomyces*, isolated from different cone snails, produce the same subset of antifungal PTMs is intriguing, and led us to speculate an ecological and evolutionary relationship between *Conus* and their *Streptomyces*. To gain insights into this we sequenced the genomes of five cone snail-associated *Streptomyces* isolates, and used a phylogenomic approach to establish taxonomic relationships, with a view to reconstruct the evolution of the PTM biosynthetic pathway.

**FIGURE 4:**
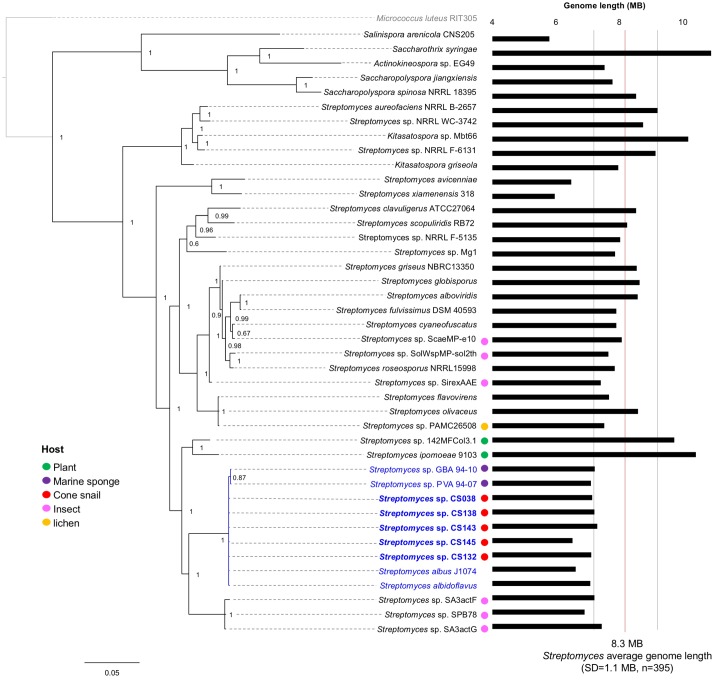
Taxonomic relationships of Actinobacteria with PTM BGCs. The phylogeny was reconstructed using rpoB, and *Micrococcus luteus* was used as root. Posterior probability values are shown at the nodes. Organisms closely related to *Streptomyces albus* are highlighted in blue, including our *Conus*-associated strains (shown in bold letters). If reported, association with eukaryotic hosts is indicated with colored circles. The bars at the right represent the genome length for each taxon; a red line indicates the average genome length for *Streptomyces* species; and the flanking gray lines show its standard deviation. Accordingly, genomes smaller than 7.2 Mbp as those from insect-, sponge- and cone snail-associated streptomycetes are considered significantly reduced and genetically related.

Preliminary analysis of the 16s rRNA sequence amplified from our isolates indicated a close genetic association with *Streptomyces albus* J1074, a terrestrial strain with a long-standing laboratory history, which is known to produce PTMs and has been exploited as a heterologous host due to its limited biosynthetic repertoire and small genome size (Olano et al., 2014). The taxonomic relationships of our strains were further investigated with an emphasis on PTM producer strains whose genomes were available, using the rpoB molecular marker, which provides better resolution for the genus *Streptomyces* than the 16s rRNA (**Figure 4**). Our phylogenetic analysis strongly suggests that all cone snail-associated *Streptomyces*, irrespective of the *Conus* species, are closely related and cluster in the same clade with the previously reported sponge-associated strains GVA94-10 and PVA 94-07 (Ian et al., 2014) and with *Streptomyces albus* J1074 (Zaburannyi et al., 2014).

Interestingly, all these *Streptomyces* are known to produce PTMs and have reduced genomes compared with the average *Streptomyces* genome length (8.3 Mbp), with a standard deviation of 1.1 Mbp as calculated from 395 publically available *Streptomyces* genomes. Consistently, the genomes of our five *Streptomyces* isolates include identical PTM BGCs and reduced genomes with an average genome length of 7 Mbp (**Figure 4**). Overall, these observations suggest a taxonomic specificity, genome reduction and a conserved PTM BGCs, as common traits in marine host-associated *Streptomyces* (**Figure 4**).

### Evolutionary Reconstruction of the PTM Biosynthetic Pathway

Given the widespread presence of the PTM BGCs in host-associated *Streptomyces*, we decided to study BGC evolution in more detail. Using a phylogenomic approach, we identified 134 PTM BGCs (Supplementary Figure S6), from which a subset of 31 BGCs was selected for having known products and representing diverse taxonomic groups. Our analyses show that the PTM BGC is conserved and widespread within Actinobacteria, and that the three members of the Gammaproteobacteria class included in the matrix, namely, *Lysobacter gummosus*, *L. enzymogenes*, and *Saccharophagus degradans*, seem to have acquired the biosynthetic capacity to produce PTMs (maltophilin-*related*), most likely through horizontal gene transfer from Actinobacteria. Moreover, assuming that all PTM chemical variants are the result of sequence divergence within their BGCs, we established direct correlations between sequence phylogeny, the presence/absence of enzymes within the BGC, and the chemical structures of PTMs produced by these biosynthetic systems. Our analyses included both the data obtained in this study for the cone snail-associated strains, and that available in the literature (Zhang et al., 2016; Saha et al., 2017).

**FIGURE 5:**
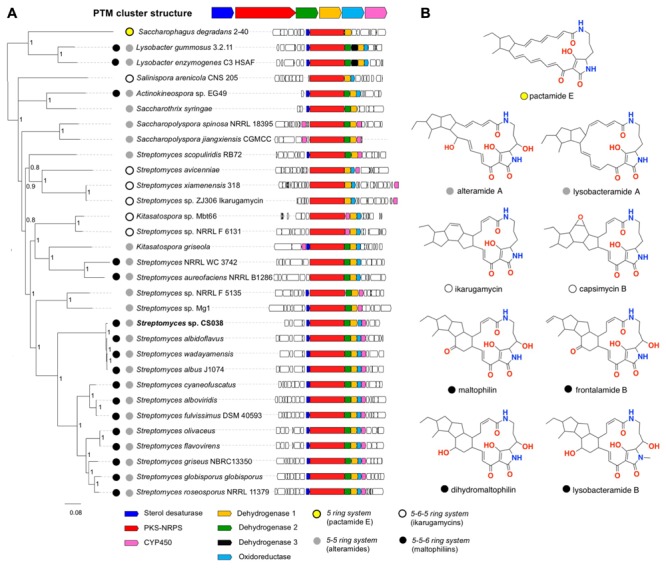
Phylogenomic analysis of the PTM BGC. **(A)** The PTM BGCs from selected genomes are sorted after the NRPS-PKS phylogeny at the left. The posterior probability values are shown at the nodes of the tree. *Streptomyces* sp. CS038, a cone snail-associated isolate, is highlighted in bold letters. Its sequence represents the conserved PTM BGCs of all cone snail-associated streptomycetes reported in this work. Predicted ring composition is indicated for each BGC with colored circles. **(B)** Selected examples of PTMs based on their ring systems.

**FIGURE 6:**
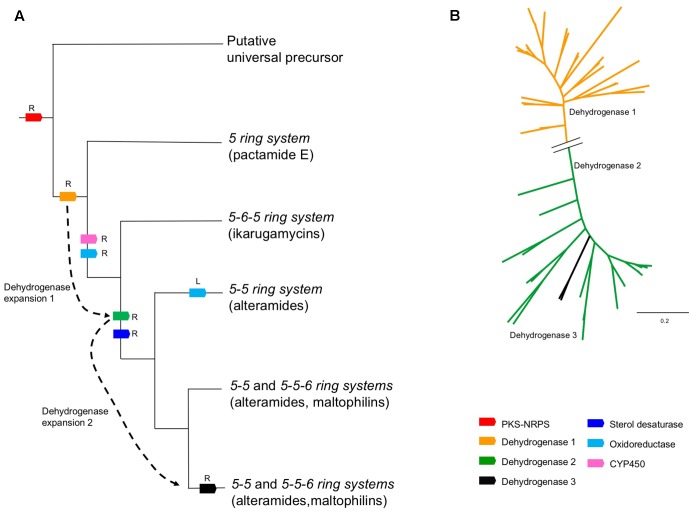
Biosynthetic evolution of PTMs. **(A)** Reconstruction of the evolutionary events leading to the chemical diversity of PTMs. The branches of the cladogram show the products of each evolutionary event. Gene recruitment events are indicated with an R, and gene losses with an L. Dotted arrow indicate expansions of the PTM dehydrogenases gene families, which led to major changes in ring composition in PTMs. **(B)** Phylogenetic reconstruction of PTM dehydrogenases. A broken line in the tree indicates the distant relationship between dehydrogenases in clades 1 and 2. Recently evolved family 3 is likely derived from a duplication event in family 3.

We found that the PTM BGCs are highly conserved, and given the current dataset, the enzymatic repertoire of the family includes only 7 enzymes: a PKS-NRPS hybrid system, a sterol desaturase (SD), a cytochrome P450 (CYP450), an oxidoreductase (OxR) and 3 dehydrogenases, named dehydrogenase 1 (DH1), 2 (DH2), and 3 (DH3), for simplicity (**Figure 5A**). We also found that the hybrid PKS/NRPS and DH1 are the only genes that are universally conserved. To a lesser extent, the OxR is highly conserved, as the only BGCs lacking this gene are those from *Saccharothrix syringae*, *Saccharopolyspora spinosa*, *Saccharopolyspora jianaxiensis*, plus three members of the *Streptomyces* genus (*Streptomyces* sp. NRRL F5135, *Streptomyces* sp. Mg1 and *Streptomyces scopuliridis*) (**Figure 5A**). OxR has been linked to the formation of the inner-membered ring, either in PTMs with three or two rings. A total of eleven BCGs analyzed lack the SD coding gene. SD is responsible for the addition of a hydroxyl group at the C-4 (see **Figure 3** for the numbering system) in the latest stage of the pathway. The cytochrome P450 coding gene is also a well-conserved gene, only missing in *Salinispora arenicola*, *Actinokineospora* sp., *Saccharothrix syringae* and two members of the *Streptomyces* family (*Streptomyces* sp. NRRL WC 3742 and *S. aureofaciens*) (**Figure 5A**). Within this context, it is interesting to note that the strains isolated from cone snails show the most conserved and widespread PTM BGC, encoding for the hybrid PKS/NRPS, sterol desaturase, DH 1 and DH 2, oxidoreductase and cytochrome P450 genes (**Figure 5A**). The reconstruction of the evolutionary events that lead to the PTM chemical diversity is shown in **Figure 6**.

## Discussion

Prior to addressing our experimental data, it is first necessary to comment on PTM scientific literature, and in particular ambiguities and redundancies associated with the assignment of chemical structures, trivial nomenclature and scaffold numbering schemes. The structure elucidation of any natural product is critically dependent on the isolation of a pure sample of sufficient quality and size to enable the acquisition and analysis of spectroscopic data (i.e., NMR, MS, [α]_D_, CD, UV-vis). Such an analysis must unambiguously assign the planar structure, enabled by the use of a consistent and informative carbon skeleton (scaffold) numbering scheme. With a definitive planar structure in hand, structure elucidation can advance to assigning relative and then absolute configurations. New natural products are typically allocated a unique trivial name, while re-isolated known natural products retain the prior-published trivial nomenclature. Where a class of natural products (i.e., PTMs) is isolated by multiple researchers, from varying sources, at different times, it is best practice to use of a consistent (common) scaffold numbering scheme and related trivial nomenclature. Unfortunately, the published history of bacterial PTMs diverges from best practice, with competing (even redundant) trivial nomenclatures and numbering schemes, and on occasion inadequate levels of spectroscopic characterisation. Consider the historical summary outlined below.

### Maltophilins (Xanthobaccins, HSAFs, Lysobacteramide B, Frontalamides, FIs, Pactamides)

A planar structure for maltophilin was first reported in 1996 from *Stenotrophomonas maltophilia* R3089 (Jakobi and Winkelmann, 1996). In 1999 maltophilin was re-isolated from *Stenotrophomonas* sp. strain SB-K88, at which time it was assigned a partial relative configuration, and designated with the additional trivial name xanthobaccin A (Hashidoko et al., 1999). Although these same authors went on to acknowledge co-metabolites xanthobaccins B and C (Nakayama et al., 1999; Hashidoko et al., 2000), these structure assignments were not validated by spectroscopic characterization. Dihydromaltophilin was first reported in 1997 from a *Streptomyces* sp., as a co-metabolite with maltophilin (Graupner et al., 1997). Although both co-metabolites were assigned partial relative configurations consistent with earlier reports, they were also designated the additional trivial names A90931a and A90931b. To add to the confusion, in 1999 dihydromaltophin was re-isolated from *Xanthomonas* sp KB-K88 (Nakayama et al., 1999), and designated the trivial name xanthobaccin B, and in 2007 was re-isolated from *L. enzymogenes* strain C3 (Yu et al., 2007), and designated the trivial name heat-stable antifungal factor (HSAF). This confusion was compounded by inconsistent use of carbon (scaffold) numbering, and its incorporation into subsequent trivial nomenclature. For example, in 2012 and 2015 follow-up studies, *L. enzymogenes* strain C3 was also reported to yield 3-deOH-HSAF (Li et al., 2012), and lysobacteramides A and B (Xu et al., 2015), respectively. Of note this latter report successfully assigned an absolute configuration to dihydromaltophilin. In 2010 the biosynthetically related frontalamides A and B, and three biosynthetic intermediates FI-1, FI-2 and FI-3 were isolated from *Streptomyces* sp. SPB78 (Blodgett et al., 2010), however, it is important to note that none of these structure assignments were supported by an adequate level of spectroscopic characterization or data analysis. In 2017 pactamides A-F were reported from the marine-derived *Streptomyces pactum* SCSIO 02999 (Saha et al., 2017). The proliferation of trivial nomenclature and numbering systems, and inadequately characterized and documented PTM structures is unfortunate. This report acknowledges the published trivial nomenclature (except where it is redundant), and employs the numbering scheme of Graupner et al. (1997) (see **Figure 3**). We also group the maltophilins, xanthobaccins, HSAFs, lysobacteramide B, frontalamides, FIs, and pactamides A-B, D and F in a common *5-5-6 ring system* category (collectively known as maltophilins) (**Figure 5B**), based on the fact they share a common carbocyclic ring system embedded within the PTM scaffold.

### Alteramide A (Aburatubolactam A, Lysobacteramide A, Pactamide C)

Alteramide was first reported in 1992 from a marine sponge-associated *Alteromonas* sp. (Shigemori et al., 1992), while the closely related aburatubolactam A was reported in 1996 from the marine mollusk-associated *Streptomyces* sp. SCRC-A20 (Bae et al., 1996). We group alteramide A, aburatubolactam A, lysobacteramide A, and pactamide C in a common *5-5 ring system* category (collectively known as alteramides) (**Figure 5B**).

### Ikarugamycins (Butremycin, Capsimycins)

The planar structure for ikarugamycin was first reported in 1977 from *Streptomyces phaeochromogenes* var. *ikaruganensis* Sakai (Ito and Hirata, 1977), and its absolute configuration confirmed by total synthesis (Pacquette and Macdonald, 1989). Butremycin, a hydroxylated ikarugamycin, was reported in 2014 from a mangrove river sediment-derived *Micromonospora* sp. K310 (Kyeremeh et al., 2014), while isoikarugamycin, 28-*N*-methylikarugamycin and 30-oxo-28-*N*-methylikarugamycin were reported in 2015 as co-metabolites with ikarugamycin from a marine sediment-derived *Streptomyces zhaozhouensis* CA-185989 (Lacret et al., 2015). In 2003 ikarugamycin epoxide was reported as a co-metabolite with ikarugamycin (and ripromycin) from *Streptomyces* sp. Tü 6239 (Bertasso et al., 2003). Capsimycin was first reported in 1979 from *Streptomyces* sp. C 49–87 (Aizawa et al., 1979), and later in 2017 as a co-metabolite with capsimycins B-G from a mangrove-derived *Streptomyces xiamenensis* 318 (Yu et al., 2017), with this latter report renaming ikarugamycin epoxide as capsimycin B. We group ikarugamycins, butremycin, and capsimycins in a common *5-6-5 ring system* category (collectively known as ikarugamycins) (**Figure 5B**).

### Pactamide E

In 2017 pactamides A–F were reported from the marine-derived *Streptomyces pactum* SCSIO 02999 (Saha et al., 2017). Building on our categories outlined above, we acknowledge pactamide E as the sole known exemplar of a *5-ring system* category of PTM (**Figure 5B**).

The PTM biosynthetic potential of *Streptomyces* sp. CMB-CS038 was studied by cultivation in different culture conditions and media, generating profiles comparable to other host-associated *Streptomyces* and with a generally very low level of production under standard culture conditions. We determined that ISP-4 agar (inorganic salt starch media) was optimal for the production of PTMs, with slower growth and a lack of PTMs production occurring in low salt media (Supplementary Figure S1). This is consistent with previous studies of marine adaptation in *Streptomyces albus* and related strains (Ian et al., 2014).

The genomes of cone snail-derived *Streptomyces* were sequenced and revealed a reduction in genome size, and share a common clade taxonomically with other host-associated *Streptomyces*. This observation is in agreement with the fact that symbiotic (or associated) microorganisms display reduced genomes (McCutcheon and Moran, 2012). For example, two *Streptomyces* (*S.* sp. PVA 94-07 and *S.* sp. GBA 94-10) associated with sponges, which have been identified as closely related species to *Streptomyces albus* J1074, have a minimized genome of 6.8 Mb (Ian et al., 2014).

In addition to taxonomic specificity, we speculated that PTMs may deliver an ecological advantage in providing anti-infective protection against fungal pathogens. There are several observations that support this view. Firstly, despite routinely isolating marine-derived fungi from all marine substrates examined in our laboratory (e.g., sand, molluscs, fish, and algae), the cone snail samples examined during this study were remarkably deficient in fungal species. Secondly, literature accounts note the PTM alteramide very likely plays a key role in keeping coral reefs free of fungal pathogens (Moree et al., 2014). Finally, our biological activity assays correlate with previous observations of dihydromaltophilin (Graupner et al., 1997), and confirm significant antifungal activity.

We based the evolutionary reconstruction of PTM biosynthetic pathway on a relatively straightforward concept: all PTM BGC structural variations must be encoded in the genome, thus, a deep look into the phylogeny based in the key conserved enzymes will directly highlight the genomic traits for a given chemical structure variation. For instance, we found that the hybrid PKS/NRPS and DH 1 constitute the core of the PTM BGC and potentially the minimal biosynthetic unit to form PTMs. This is supported by previous studies showing that DH 1 is essential for the formation of the *5-ring system* (Saha et al., 2017), which prompts us to predict involvement of the cryptic BGC found in *Saccharophagus degradans* 2–40.

In addition, the analysis shown indicates that the presence/absence of the dehydrogenase homologs determines the PTM ring composition (**Figure 6A**). This assumption was supported upon a dehydrogenase-based phylogenetic analysis that shows a remote expansion event leading to the evolution of DH1 and DH2, and a recent duplication event (DH3) (**Figure 6B**). Therefore, BGCs with the combo DH1/oxidoreductase will produce an ikarugamycins *5-6-5 ring system* (**Figure 6A**). The presence of the DH 1 and DH 2 correlates with the alteramides *5-5 ring system* (**Figure 6A**). Finally, DH 1, DH 2 and the oxidoreductase will assemble a maltophilins *5-5-6 ring system* (**Figure 6A**). While the lack of an oxidoreductase homolog is exclusive for the formation of a *5-5 ring system*, phylogenomics strongly suggests that bacteria producing the *5-5-6 ring system* can additionally produce a *5-5 ring system*. For example, the *epi*-alteramide BGC produced by *Streptomyces albus* J1074 harbors DH 1, DH 2 and an oxidoreductase (Olano et al., 2014). On a chemical level, both lysobacteramide A (5-5 *ring system*) and lysobacteramide B (5-5-6 *ring system*) have been isolated from *L. enzymogenes* (Xu et al., 2015), which is consistent with our hypothesis. Consistent with this analysis, we identified the production of four PTMs (**1**–**4**) from *Streptomyces* sp. CMB-CS038, all displaying a *5-5-6 ring system* (**Figure 3**).

Our phylogenetic analysis also suggests that *Lysobacter* sp. acquired, most likely from an actinobacterium, the maltophilins BGC via horizontal gene transfer – capable of producing both maltophilin and dihydromaltophilin. *Lysobacter* PTM BGCs are unique as they include an extra dehydrogenase homolog (DH 3), which to the best of our knowledge, has not been linked to a particular function (**Figure 6A**). *Lysobacter* PTM BGCs also need to accommodate lysobacteramide B which features an *N*-methylated tetramic acid *moiety*. Regarding the sterol desaturase, our analysis was consistent with previous experimental evidence showing that the presence of this enzyme correlates with C-4 hydroxylation (see **Figure 3** for the numbering system). In addition, our study shows that ikarugamycins lack C-4 hydroxylation step, and the related BGCs lack a sterol desaturase. Finally, activity of the cytochrome P450 homolog (CYP450) has been recently demonstrated through gene knock-out and complementation experiments in a capsimycin producing *Streptomyces* sp. Cytochrome P450 activity is responsible of an epoxide ring formation in the capsimycin and capsimycin B, and further side chain hydroxylation in capsimycin G (Yu et al., 2017).

Altogether, our combined chemical, bioactivity and phylogenomic analysis shows that, (i) there is a taxonomic bias of isolated cone snail-associated *Streptomyces* toward other host-associated *Streptomyces* species; (ii) this bias is reflected at a genomic level (i.e., common reduced genome), and biosynthetic level (i.e., common PTM BGCs), and (iii) there is an underlying antifungal property for PTMs that offers an ecological (survival) advantage. Our investigation also highlights the importance of combining natural products and analytical chemistry, next generation sequencing, and phylogenomic analysis, to achieve a better understanding of natural product biosynthesis, ecology and evolution. Specifically, our work emphasizes how metabolic pathway evolution within its ecological and taxonomic context can provide interesting mechanistic hypotheses pinpointing specific genetic targets responsible of structural modifications.

## Author Contributions

MQ designed experiments, performed microbiological isolations, performed and analyzed spectroscopic and analytical experiments and wrote the manuscript. CL-C performed genomic analysis, phylogenomic analysis and wrote the manuscript. PC-M performed and analyzed phylogenomic analyses and genome mining and wrote the manuscript. AS performed analytical experiments, analyzed spectroscopic data and wrote the manuscript. EM analyzed genomic data and wrote the manuscript. FB-G analyzed genomic and phylogenomic data and wrote the manuscript. RC designed the analytical experiments, analyzed the analytical data and wrote the manuscript.

## Conflict of Interest Statement

The authors declare that the research was conducted in the absence of any commercial or financial relationships that could be construed as a potential conflict of interest.

## Footnotes

1 http://biology.burke.washington.edu/conus/catalogue/index.php

2 https://github.com/nselem/EvoDivMet/tree/master/CORASON
