# Supplementary material for: Diverse Cone-Snail Species Harbor Closely Related Streptomyces Species with Conserved Chemical and Genetic Profiles, Including Polycyclic Tetramic Acid Macrolactams
Source: Front Microbiol. 2017 Nov 24;8:2305. doi: 10.3389/fmicb.2017.02305 (PMC5705629; doi:10.3389/fmicb.2017.02305)
Supplement: Supplementary file 1 [file Table_1.PDF]

Supporting information

Diverse cone-snail species harbor closely related *Streptomyces* species with conserved chemical and genetic and profiles, including polycyclic tetramic acid macrolactams

Michelle Quezada[§,a], Cuauhtemoc Licona-Cassani[§,b,c], Pablo Cruz-Morales[b], Angela A. Salim[a], Esteban Marcellin[d], Robert J. Capon[a*] and Francisco Barona-Gomez[b*]

[a]Institute for Molecular Bioscience, The University of Queensland, Brisbane, QLD, Australia; [b]Evolution of Metabolic Diversity Laboratory, Unidad de Genómica Avanzada (Langebio), Cinvestav-IPN, Irapuato, México; [c] Centro de Biotecnología-FEMSA, Tecnológico de Monterrey, Monterrey, NL, México; [d]Australian Institute for Bioengineering and Nanotechnology, The University of Queensland, Brisbane, QLD, Australia.

[§]Authors contributed equally to the manuscript. [*]Corresponding authors.

Correspondence:
Robert J. Capon
Institute for Molecular Bioscience, The University of Queensland, Brisbane, QLD, Australia
Email: r.capon@uq.edu.au

Francisco Barona-Gomez
Evolution of Metabolic Diversity Laboratory, Unidad de Genomica Avanzada (Langebio), Cinvestav-IPN, Irapuato, México
Email: francisco.barona@cinvestav.mx

Table of contents

Characterisation of compounds **1-4** 4

Table S1. Cone snail-derived *Streptomyces* sp. isolates 3
Table S2. Comparison of NMR (600 MHz) data for **1** and dihydromaltophilin[a] 5

Figure S1. Picture of *Streptomyces* sp. (CMB-CS038) cultivated in ISP-4, Marine and Nutrient (without salt) agar 3
Figure S2. ^{1}H NMR (600 MHz, MeOH-d_4) spectrum of **1** 6
Figure S3. 2D NMR (600 MHz, MeOH-d_4) key correlations for **1** 6
Figure S4. HSQC NMR NMR (600 MHz, MeOH-d_4) spectrum of **1** 7
Figure S5. Experimental ECD spectra of **1** in MeOH 7
Figure S6. PTM Biosynthetic Gene Clusters 8
Figure S7. Antifungal assay for **1** against C. albicans (ATCC 90028) 10
Figure S8. Cytotoxicity activity of **1** against SW-620 (human colon cancer) and NCI-H460 (lung cancer) 10

Table S1. Cone snail-derived *Streptomyces* sp. isolates

Strain code	Cone snail (species)	Tissue
CMB-CS138	*Conus ebraeus*	hepatopancreas
CMB-CS145	*Conus flavidus*	stomach
CMB-CS143	*Conus coronatus*	stomach
CMB-CS038	*Conus miles*	stomach
CMB-CS132	*Conus emaciatus*	hepatopancreas

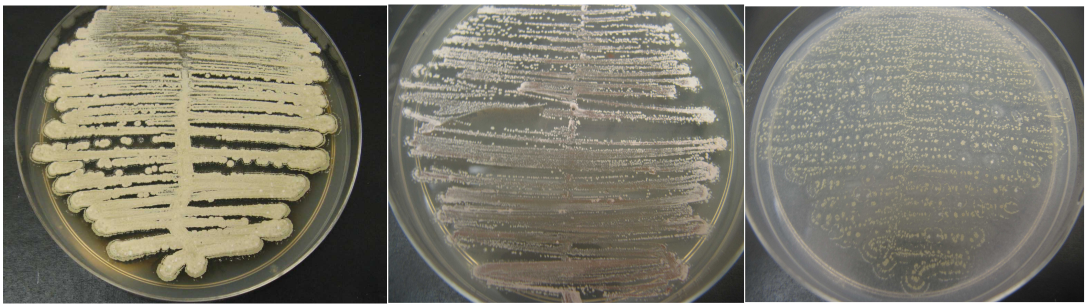

ISP4 Marine Nutrient agar

Figure S1. Picture of *Streptomyces* sp. (CMB-CS038) cultivated in ISP-4, Marine and Nutrient (without salt) agar

Characterisation of compounds 1-4

Dihydromaltophilin (1). Colorless powder; $[\alpha]_D^{22}$ + 9.2 (*c* 0.05, MeOH); UV-vis (MeOH) λ_{max} (log ε) 271 (3.91), 306 (3.86), 371 (3.79) nm; ESI(–)MS *m*/*z* 511 $[M - H]^-$; HRESI(–)MS *m*/*z* 511.2807 $[M - H]^-$ (calcd for $C_{29}H_{39}N_2O_6^-$, 511.2814).

Δ^{30}-Dihydromaltophilin (2). ESI(–)MS *m*/*z* 509 $[M - H]^-$; HRESI(+)MS *m*/*z* 533.2630 $[M + Na]^+$ (calcd for $C_{29}H_{38}N_2O_6Na$, 533.2622).

Xanthobaccin C (3). ESI(–)MS *m*/*z* 493 $[M - H]^-$; HRESI(+)MS *m*/*z* 517.2677 $[M + Na]^+$ (calcd for $C_{29}H_{38}N_2O_5Na$, 517.2673).

FI-3 (4). ESI(–)MS *m*/*z* 491 $[M - H]^-$; HRESI(+)MS *m*/*z* 515.2524 $[M + Na]^+$ (calcd for $C_{29}H_{36}N_2O_5Na$, 515.2516).

Table S2. Comparison of NMR (600 MHz) data for 1 and dihydromaltophilin[a]

	δ_C[b]	δ_H, mult (*J* in Hz)[b]	δ_C[c]	δ_H, mult[c,d]
2	37.9	a 3.49, m	38.0	a 3.48, ddd
		b 2.79, ddd (*12.8, 12.8, 1.8*)		b 2.78, ddd
3	32.4	a 1.65, m	32.3	a 1.6, m
		b 1.32, m		b 1.4, m
4	72.8	3.93, ddd (*6.4, 2.0, 2.0*)	72.4	3.93, br d
5	67.9	3.73, br s	69.0	3.85, br s
7				
8			103	
9				
10	nd[e]	7.27, br d (*14.0*)	126.8	7.19, br d
11	nd[e]	6.45, dd *(14.0, 11.0)*	148.9	6.52, dd
12	46.2	2.10, dddd (*11.0, 11.0, 11.0, 3.0*)	46.7	2.1, m
13	44.0	a 1.89, ddd (*12.8, 4.2, 3.0*)	43.3	a 1.90, ddd
		b 1.29, m		b 1.3, m
14	75.6	3.33, ddd (*10.5, 10.5, 4.2*)	75.2	3.34, ddd
15	60.9	1.18, ddd (*10.5, 10.5, 10.5*)	60.6	1.19, ddd
16	60.0	1.86, ddd (*10.5, 9.5, 2.0*)	59.7	1.85, ddd
17	48.0	1.33, m	48.1	1.3, m
18	55.6	1.36, m	55.4	1.3, m
19	41.9	a 2.08, m	41.7	a 2.07, m
		b 0.86, ddd (*11.7, 11.7, 11.7*)		b 0.94, ddd
20	43.6	2.43, m	43.3	2.43, dddd
21	38.9	a 2.09, m	38.7	a 2.07, m
		b 0.96, ddd (*12.0, 11.5, 8.5*)		b 0.94, ddd
22	49.6	1.69, dddd (*11.5, 11.5, 10.5, 5.8*)	49.3	1.67, ddd
23	45.8	1.24, m	45.4	1.3, m
24	29.8	a 3.42, m	29.6	a 3.44, ddd
		b 2.11, m		b 2.15, m
25	142.1	6.02, ddd (*11.6, 11.6, 1.5*)	142.0	6.00, ddd
26	124.8	5.80, dd (*11.6, 2.4*)	124.6	5.78, dd
27	168.5		168.7	
28	194.9		195.2	
29	19.1	1.09, d (*6.3*)	19.0	1.09, d
30	27.6	a 1.61, m	27.4	a 1.60, m
		b 1.09, m		b 1.09, m
31	13.2	0.89, t (*7.4*)	13.2	0.88, t

[a] Graupner, P.R., Thornburgh, S., Mathieson, J.T., Chapin, E.L., Kemmitt, G.M., Brown, J.M., Spines, C.E. (1997). Dihydromaltophilin: a novel fungicidal tetramic acid containing metabolite from *Streptomyces* sp. . *Antibiotics* 50(12), 1014-1019. [b] Measured in MeOH-d_4 and referenced to residual signals (δ_H 3.30 and δ_C 49.1 ppm) in deuterated solvents. [c] Measured in $CDCl_3$:MeOH-d_3 (1:3). [d] The value (Hz) of multiplicity was not reported in the publication. [e] Carbon resonance is not detected due to signal broadening.

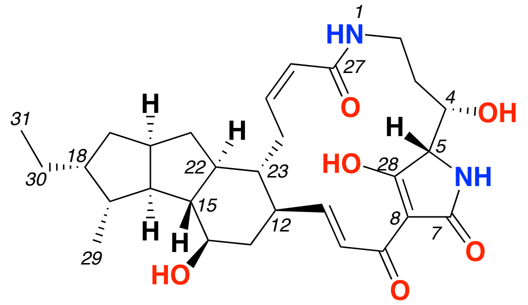

dihydromaltophilin (**1**)

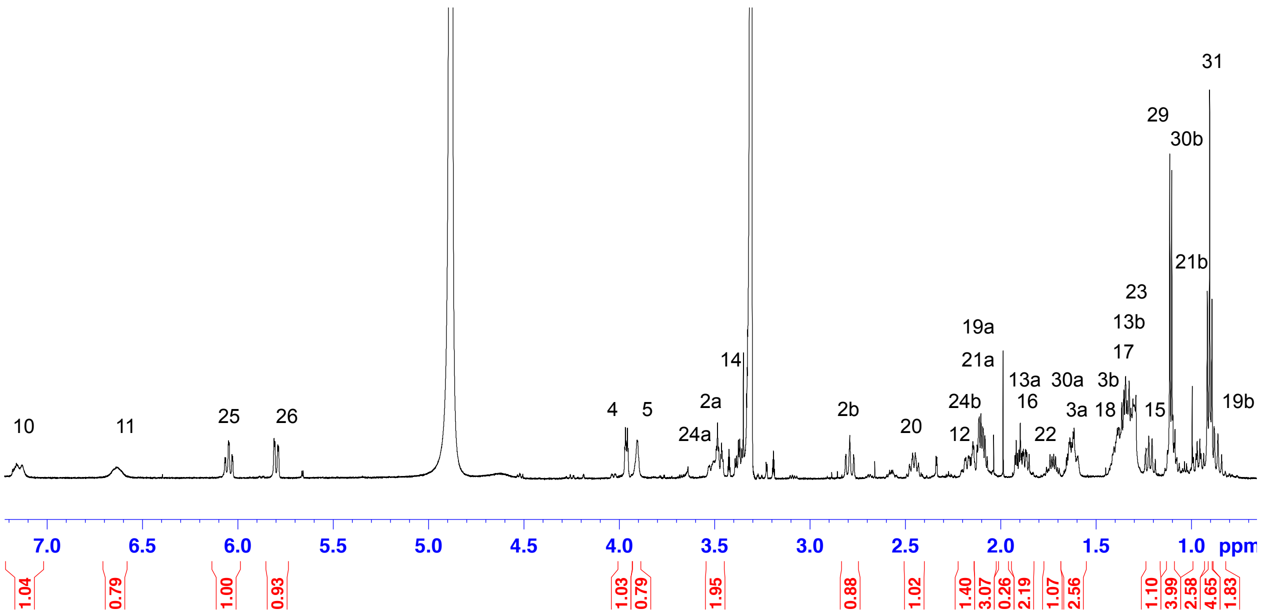

Figure S2. ^{1}H NMR (600 MHz, MeOH-d_4) spectrum of 1

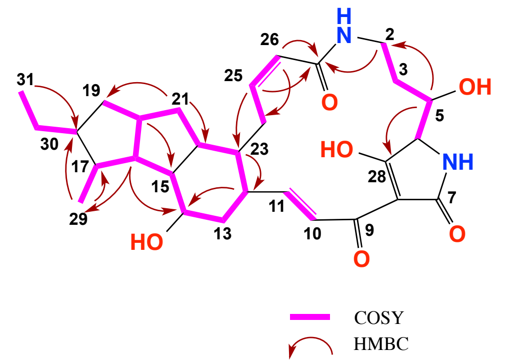

Figure S3. 2D NMR (600 MHz, MeOH-d_4) key correlations for 1

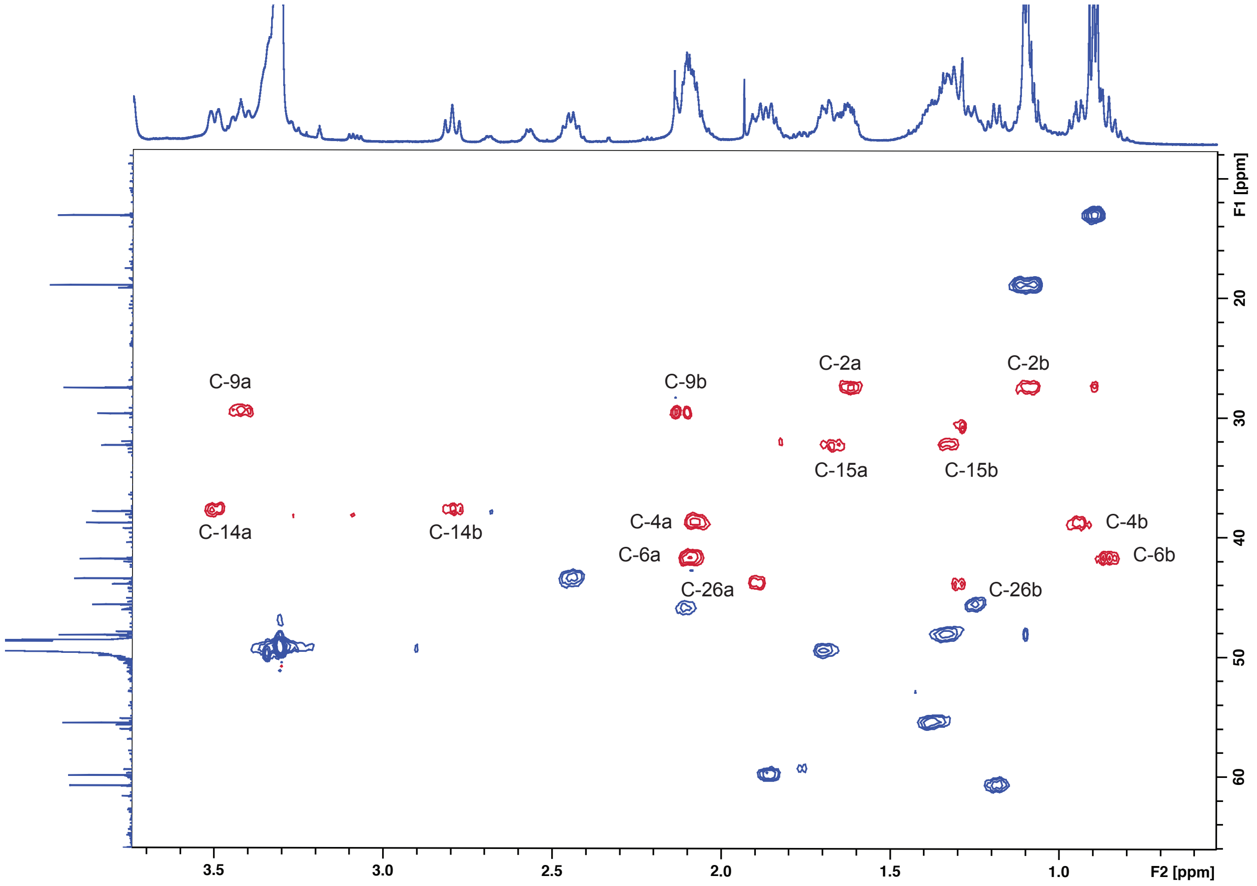

Figure S4. HSQC NMR NMR (600 MHz, MeOH-d_4) spectrum of 1

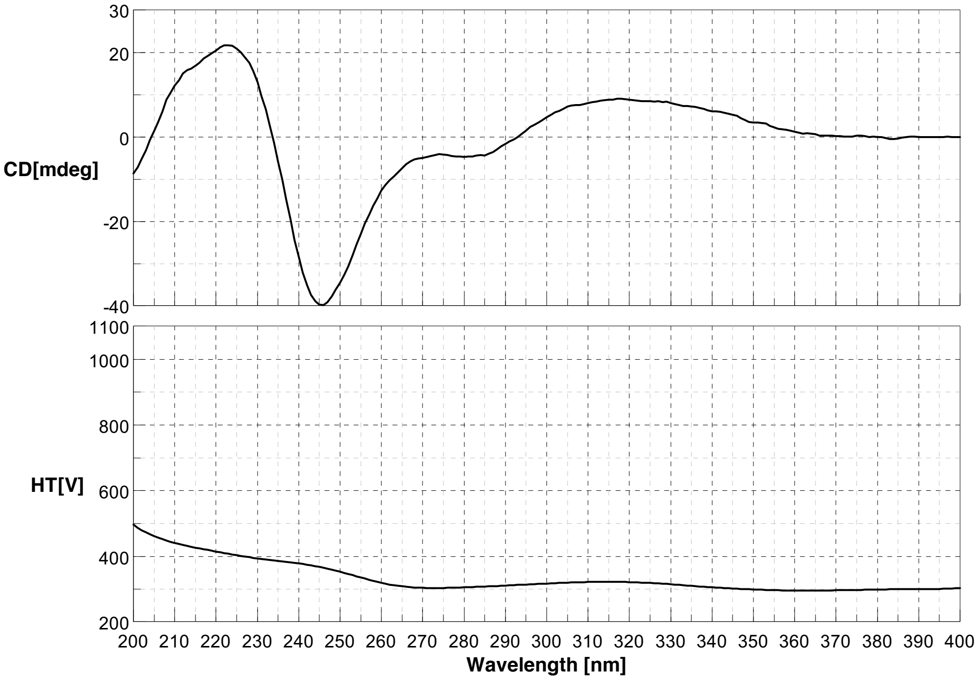

Figure S5. Experimental ECD spectra of 1 in MeOH.[a]

[a] The measured spectrum is highly similar to the ECD measurements on dihydromaltophilin (HSAF) published values in the literature (Xu, L., Wu, P., Wright, S. J., Du, L., Wei, X. (2015). Bioactive polycyclic tetramate macrolactams from *Lysobacter enzymogenes* and their absolute configurations by theoretical ECD calculations. *J. Nat. Prod.* 78, 1841-1847).

Figure S6. PTM Biosynthetic Gene Clusters

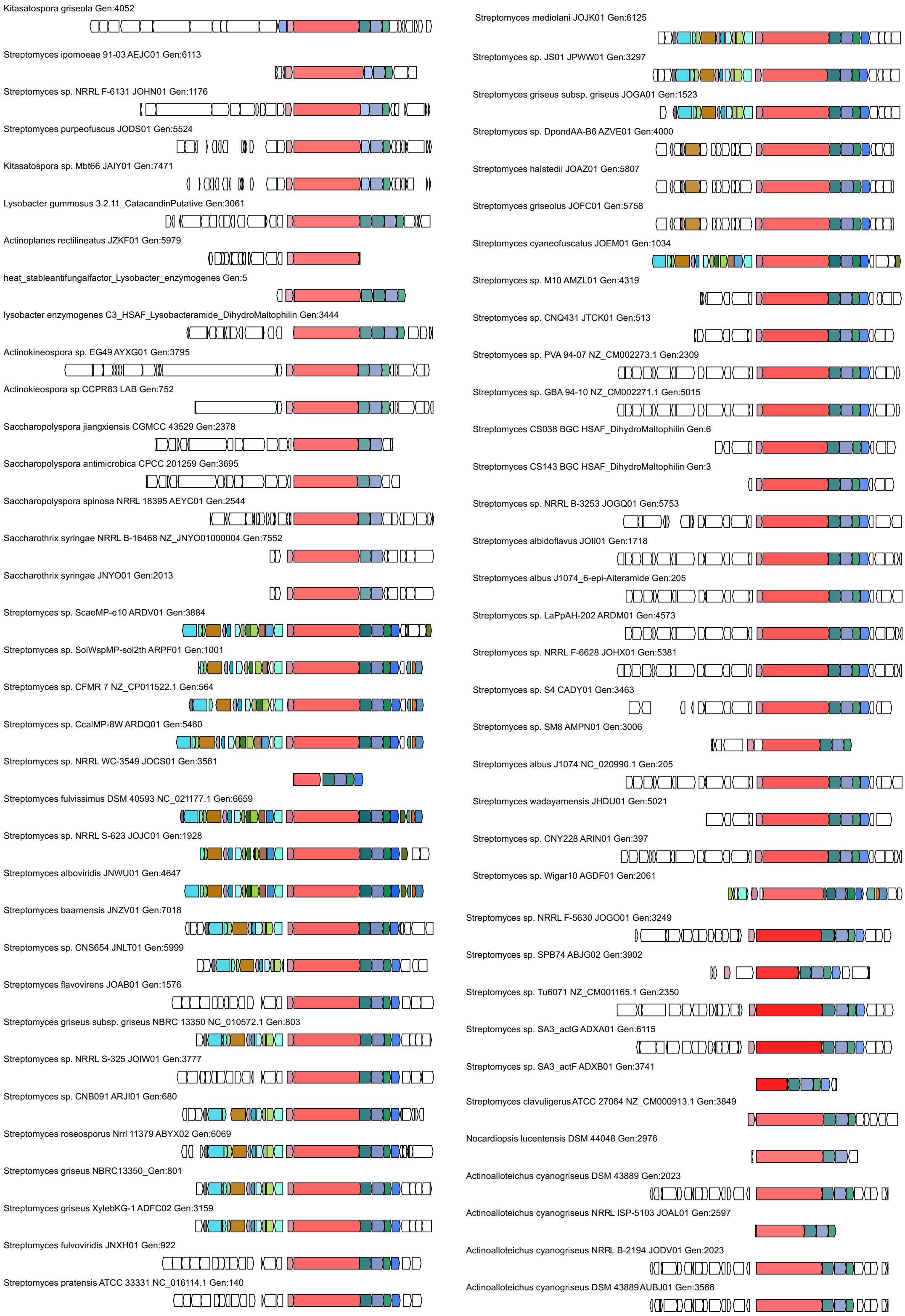

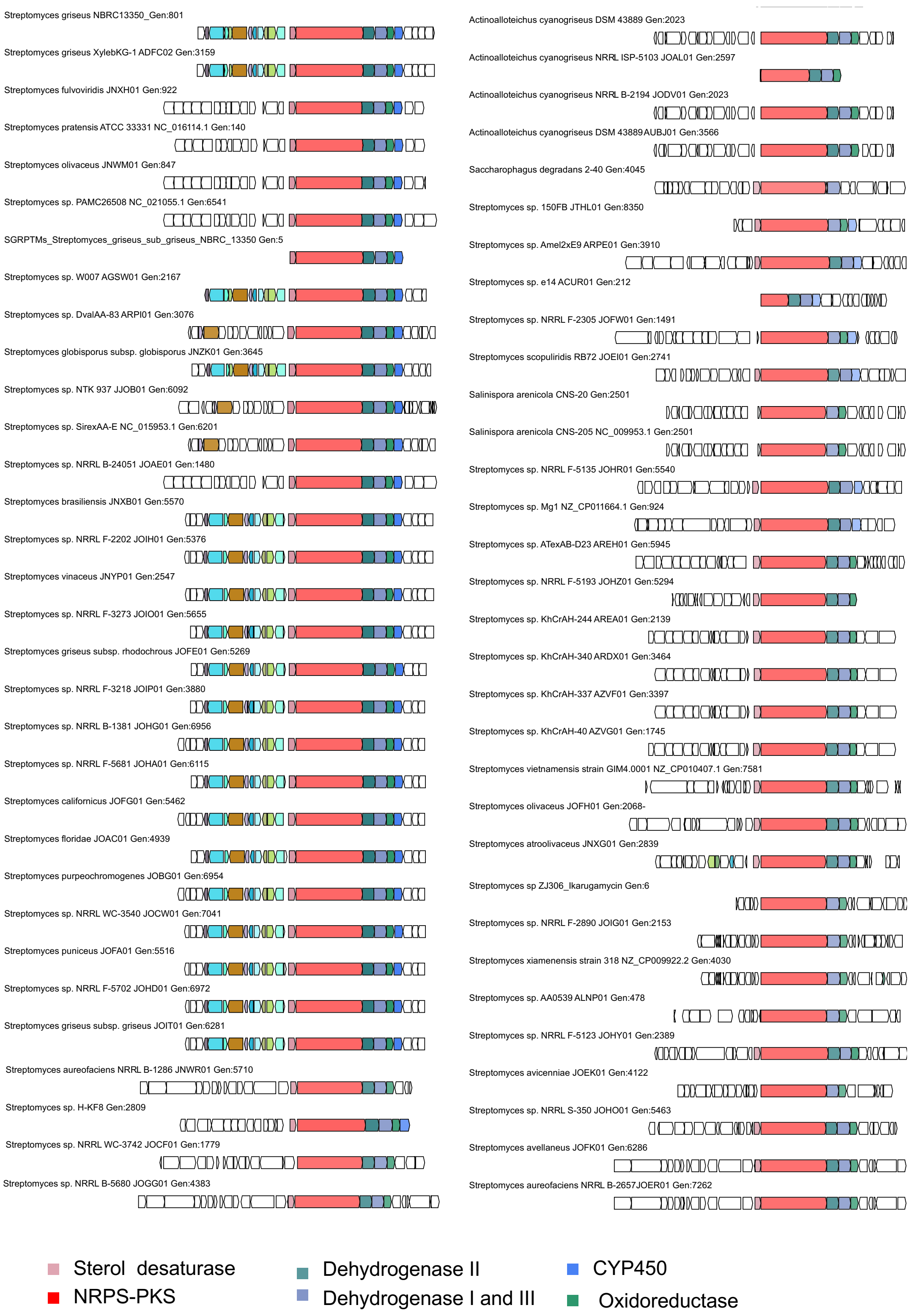

Streptomyces griseus NBRC13350_Gen:801
Streptomyces griseus XylebKG-1 ADFC02 Gen:3159
Streptomyces fulvoviridis JNXH01 Gen:922
Streptomyces pratensis ATCC 33331 NC_016114.1 Gen:140
Streptomyces olivaceus JNWM01 Gen:847
Streptomyces sp. PAMC26508 NC_021055.1 Gen:6541
SGRPTMs_Streptomyces_griseus_sub_griseus_NBRC_13350 Gen:5
Streptomyces sp. W007 AGSW01 Gen:2167
Streptomyces sp. DvalAA-83 ARPI01 Gen:3076
Streptomyces globisporus subsp. globisporus JNZK01 Gen:3645
Streptomyces sp. NTK 937 JJOB01 Gen:6092
Streptomyces sp. SirexAA-E NC_015953.1 Gen:6201
Streptomyces sp. NRRL B-24051 JOAE01 Gen:1480
Streptomyces brasiliensis JNXB01 Gen:5570
Streptomyces sp. NRRL F-2202 JOIH01 Gen:5376
Streptomyces vinaceus JNYP01 Gen:2547
Streptomyces sp. NRRL F-3273 JOIO01 Gen:5655
Streptomyces griseus subsp. rhodochrous JOFE01 Gen:5269
Streptomyces sp. NRRL F-3218 JOIP01 Gen:3880
Streptomyces sp. NRRL B-1381 JOHG01 Gen:6956
Streptomyces sp. NRRL F-5681 JOHA01 Gen:6115
Streptomyces californicus JOFG01 Gen:5462
Streptomyces floridae JOAC01 Gen:4939
Streptomyces purpeochromogenes JOBG01 Gen:6954
Streptomyces sp. NRRL WC-3540 JOCW01 Gen:7041
Streptomyces puniceus JOFA01 Gen:5516
Streptomyces sp. NRRL F-5702 JOHD01 Gen:6972
Streptomyces griseus subsp. griseus JOIT01 Gen:6281
Streptomyces aureofaciens NRRL B-1286 JNWR01 Gen:5710
Streptomyces sp. H-KF8 Gen:2809
Streptomyces sp. NRRL WC-3742 JOCF01 Gen:1779
Streptomyces sp. NRRL B-5680 JOGG01 Gen:4383
Actinoalloteichus cyanogriseus DSM 43889 Gen:2023
Actinoalloteichus cyanogriseus NRRL ISP-5103 JOAL01 Gen:2597
Actinoalloteichus cyanogriseus NRRL B-2194 JODV01 Gen:2023
Actinoalloteichus cyanogriseus DSM 43889 AUBJ01 Gen:3566
Saccharophagus degradans 2-40 Gen:4045
Streptomyces sp. 150FB JTHL01 Gen:8350
Streptomyces sp. Amel2xE9 ARPE01 Gen:3910
Streptomyces sp. e14 ACUR01 Gen:212
Streptomyces sp. NRRL F-2305 JOFW01 Gen:1491
Streptomyces scopuliridis RB72 JOEI01 Gen:2741
Salinispora arenicola CNS-20 Gen:2501
Salinispora arenicola CNS-205 NC_009953.1 Gen:2501
Streptomyces sp. NRRL F-5135 JOHR01 Gen:5540
Streptomyces sp. Mg1 NZ_CP011664.1 Gen:924
Streptomyces sp. ATexAB-D23 AREH01 Gen:5945
Streptomyces sp. NRRL F-5193 JOHZ01 Gen:5294
Streptomyces sp. KhCrAH-244 AREA01 Gen:2139
Streptomyces sp. KhCrAH-340 ARDX01 Gen:3464
Streptomyces sp. KhCrAH-337 AZVF01 Gen:3397
Streptomyces sp. KhCrAH-40 AZVG01 Gen:1745
Streptomyces vietnamensis strain GIM4.0001 NZ_CP010407.1 Gen:7581
Streptomyces olivaceus JOFH01 Gen:2068-
Streptomyces atroolivaceus JNXG01 Gen:2839
Streptomyces sp ZJ306_Ikarugamycin Gen:6
Streptomyces sp. NRRL F-2890 JOIG01 Gen:2153
Streptomyces xiamenensis strain 318 NZ_CP009922.2 Gen:4030
Streptomyces sp. AA0539 ALNP01 Gen:478
Streptomyces sp. NRRL F-5123 JOHY01 Gen:2389
Streptomyces avicenniae JOEK01 Gen:4122
Streptomyces sp. NRRL S-350 JOHO01 Gen:5463
Streptomyces avellaneus JOFK01 Gen:6286
Streptomyces aureofaciens NRRL B-2657JOER01 Gen:7262
Sterol desaturase
NRPS-PKS
Dehydrogenase II
Dehydrogenase I and III
CYP450
Oxidoreductase

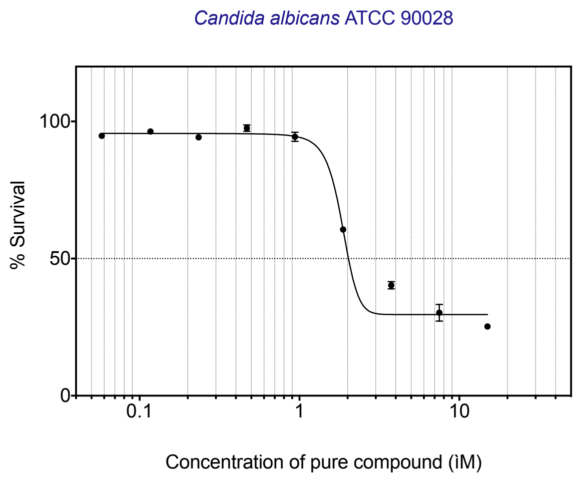

Figure S7. Antifungal assay for 1 against *C. albicans* (ATCC 90028)

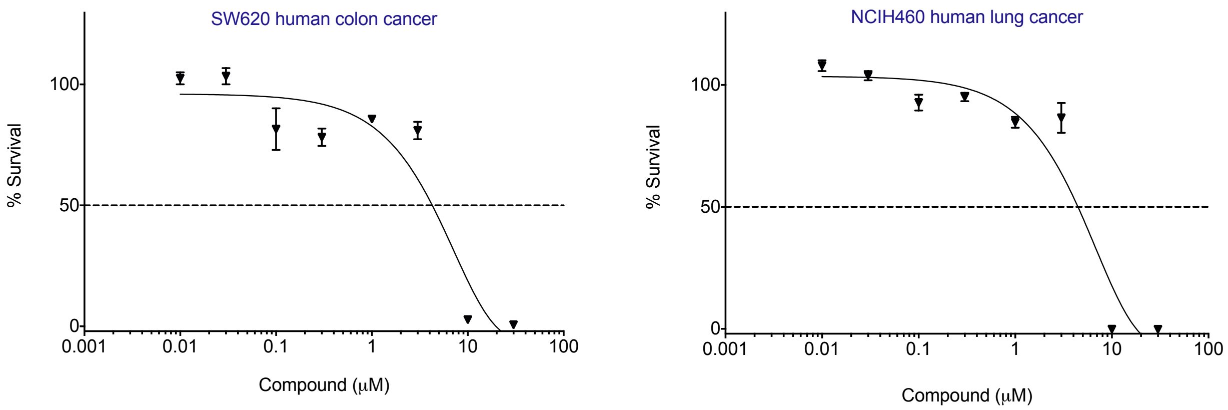

Figure S8. Cytotoxicity activity of 1 against SW-620 (human colon cancer) and NCI-H460 (lung cancer)
